# Antimicrobial Property of Polypropylene Composites and Functionalized Copper Nanoparticles

**DOI:** 10.3390/polym13111694

**Published:** 2021-05-22

**Authors:** Noemi Jardón-Maximino, Gregorio Cadenas-Pliego, Carlos A. Ávila-Orta, Víctor Eduardo Comparán-Padilla, Luis E. Lugo-Uribe, Marissa Pérez-Alvarez, Salvador Fernández Tavizón, Gerardo de Jesús Sosa Santillán

**Affiliations:** 1 Centro de Investigación en Química Aplicada (CIQA), Saltillo, Coahuila 25294, Mexico; nahomy_mimis@hotmail.com (N.J.-M.); carlos.avila@ciqa.edu.mx (C.A.Á.-O.); victor.comparan@ciqa.edu.mx (V.E.C.-P.); pamarissa@hotmail.com (M.P.-A.); salvador.fernandez@ciqa.edu.mx (S.F.T.); 2 Centro de Tecnología Avanzada CIATEQ, Lerma, Estado de México 542004, Mexico; luis.lugo@ciateq.mx; 3 Facultad de Ciencias Químicas, Universidad Autónoma de Coahuila, Saltillo, Coahuila 25280, Mexico; gdejsosa@uadec.edu.mx

**Keywords:** antimicrobial, metallic ions, ligand, copper nanoparticles, polypropylene

## Abstract

Copper nanoparticles (CuNPs) functionalized with polyethyleneimine (PEI) and 4-aminobutyric acid (GABA) were used to obtain composites with isotactic polypropylene (iPP). The iPP/CuNPs composites were prepared at copper concentrations of 0.25–5.0 wt % by melt mixing, no evidence of oxidation of the CuNP was observed. Furthermore, the release of copper ions from iPP/CuNPs composites in an aqueous medium was studied. The release of cupric ions was higher in the composites with 2.5 and 5.0 wt %. These composites showed excellent antibacterial activity (AA) toward *Pseudomona aeruginosa* (*P. aeruginosa*) and *Staphylococcus aureus* (*S. aureus*). The incorporation of CuNP into the iPP polymeric matrix slightly decreased the thermal stability of the composite material but improved the crystallinity and the storage modulus. This evidence suggests that CuNPs could work as nucleating agents in the iPP crystallization process. The iPP/CuNPs composites presented better AA properties compared to similar composites reported previously. This behavior indicates that the new materials have great potential to be used in various applications that can be explored in the future.

## 1. Introduction

From an academic and industrial viewpoint, the synthesis of CuNPs has been of great interest due to their potential applications. Examples of these include the production of conductive and antibacterial materials and as a replacement of expensive materials, such as gold and silver nanoparticles [1,2]. The antimicrobial properties of copper have been well known for a long time. Recently, copper was considered the first and only metal with antimicrobial properties by the American Environmental Protection Agency (EPA) [3]. This material eliminates 99.9% of most pathogens within 2 h of contact [4]. Furthermore, in some specific cases, copper has better properties compared to other expensive metals with antimicrobial activity, such as silver and gold [5].

Recently, several research works have emphasized the importance of CuNPs in priority areas for society such as medicine (COVID-19 pandemic) [6,7], agriculture [8,9,10,11], and environment [12,13], among others.

The addition of CuNPs into iPP has given excellent results for the inhibition of the growth of a broad microorganism spectrum. Applications of such polymeric composites include among the most important ones’ food packaging, medical instruments, and water treatment [14,15,16].

Copper/polymer composites show bactericide properties that can be attributed to their ability to release metal ions in an aqueous medium [14]. The metal ions promote an electrostatic interaction with the negatively charged bacterial cell wall, disrupting its integrity to the point of rupture. This behavior leads to leakage of intracellular material and subsequent cell death [17,18,19,20,21,22,23].

The release process of the metal ions from the composites starts with water diffusion into the composite bulk. This is followed by the reaction between metallic particles and water molecules that leads to the formation of metal ions. Finally, the migration of these metal ions towards the external surface of the composite allows its interaction with bacteria [14,24].

The properties of the polymeric matrix, such as crystallinity and hydrophobic behavior, may influence the capability of the composite to release metal ions [14,25]. Damm et al. argued that the diffusion of water molecules and metal ions occurs in the amorphous regions of the polymer matrix, then an increment in the hydrophilicity and decrease of the polymer matrix crystallinity could improve the release of ions [26].

Nonetheless, an important problem that can be observed frequently in composite materials obtained by melt mixing is the poor dispersion of the nanoparticles (NPs) in the polymeric matrix. The aggregation of NPs in a polymeric matrix is associated with its high surface energy. Typically, the formation of large NP aggregates involves a decrease in the mechanical, thermal, and antimicrobial properties of the composite [27].

On the other hand, the compatibility of the composite components can influence too on the aggregation of metallic NPs inside the polymeric matrix. Some strategies applied to improve the CuNPs compatibility with the polymeric matrix consist of functionalization of the NPs and dispersion by physical methods such as ultrasound [28]. A highly feasible option contemplates the functionalization of the CuNPs with ligands that contain both polar and non-polar moieties in their chemical structure. The polar moiety could interact with the CuNPs, whereas the non-polar moiety could do it with the polypropylene matrix. Yurong Wu. et al. obtained by solution mixing composites formed by iPP and Cu_2_O NPs modified by silanes [28]. Ramazanov et al. produced composites of iPP and functionalized CuNPs with sodium oleate through a solution mixing process [29]. Palza et al. obtained by a melt mixing method composites formed by iPP and CuNPs functionalized by hexanethiol [30]. Molaba et al. developed a method that includes, as the first step, the mixing of silver nanoparticles (AgNPs) with a paraffinic wax followed by melt blending with iPP [31]. In all these cited works it was reported good dispersion of the CuNPs in the polymeric matrix. However, the non-polar moiety of the ligand coating the NPs could affect the release process of the metal ions from the composite in an aqueous medium.

Our research group has reported the obtention of functionalized CuNPs with nitrogenated ligands [32,33,34]. These modified CuNPs were employed successfully to prepare polymeric composites with nylon 6, achieving an improvement in the mechanical properties of this polymer with a low concentration of the copper nanoparticles [27]. It is expected that these composites exhibit good antimicrobial properties due to the combination of the polar nature of the polymeric matrix with the functionalized CuNPs with nitrogenated ligands. Generally, it is considered that CuNPs functionalized with polar coatings can favor the process of liberation of metal ions and the elimination of bacteria and viruses.

In this work the obtention of composites based on iPP and CuNPs functionalized with polyethyleneimine (PEI) and 4-aminobutyric acid (GABA) by a melt mixing process is reported. Several CuNP concentrations (0.25, 1.0, 2.5, and 5.0 wt %) were employed to prepare the composites. Additionally, the copper ions release was studied and its effect on the antibacterial activity toward *Pseudomona aeruginosa* and *Staphylococcus aureus*. Finally, the mechanical and thermal properties of the composites were evaluated.

## 2. Materials and Methods

### 2.1. Materials

The isotactic polypropylene (iPP) used in this study was Valtec HS013 from Indelpro S.A. de C.V. (Altamira, México), with a melt flow index (MFI) (at 230 °C with 2.16 kg) of 11 g/10 min and an average molecular weight (Mn) of 74,784 g/mol, molecular weight distribution (MWD) of 3.9. MW and MWD were determined by gel permeation chromatography using a Waters 150 °C chromatograph eluting with 1,2,4-tricholorobenzene at 135 °C. Narrow MWD polystyrene standards were used for GPC calibration. Copper nanoparticles functionalized with polyethyleneimine and 4-aminobutyric acid (CuNPs-PEI/GABA) were obtained according to a previously reported procedure developed in our research group [35]. The nanoparticles present an average particle size of 27.0 nm and a ligand content of 8 wt %.

### 2.2. Composite Preparation

iPP/CuNPs composites were prepared using an internal mixer ATR Brabender Plasticorder (C.W.B. Brabender Instrument, Inc., South Hackensack, NJ, USA) at 190 °C and 100 rpm. The iPP and different samples with CuNPs concentrations of 0.25, 1.0, 2.5, and 5.0 wt % were processed in the same way in batches of 65 g. Plates were obtained from the composites in a P.H.I. Press (P.H.I, City of Industry, CA, USA), which was operated at a temperature of 180 °C and 17.0 MPA for 10 min. After this time, the plates are kept under pressure and are cooled with water at a cooling rate of approximately 18 °C/min. The obtained plates had a thickness of 3.0 mm.

For the study of copper ions releasing and antimicrobial activity, 2 cm × 2.4 cm samples were cut from the composite’s plates and for the DMA analysis, 3.5 cm × 1.2 cm samples were prepared.

### 2.3. Characterization

Wide-angle X-ray diffraction analysis (WAXD) was employed to identify the crystalline structure of the composites. It was used a diffractometer Siemens D-5000 (SIEMENS, Berlin, Germany) operated at 35 kV and a current of 25 mA. The scan range on the 2θ scale was from 20 to 80° with a step size of 0.02°/s.

Scanning electron microscopy (SEM) was employed to observe the dispersion of the CuNPs in the polymeric matrix. A JEOL JSM-7001F electron microscope (Jeol LTD., Akishima, Tokyo, Japan) was used, applying a voltage of 8 kV, secondary electrons detector COMPO and a work distance (WD) of 8 mm. Image acquisition was performed using the backscattered electron signal detector. The samples were cryogenically broken and the fracture surfaces were electrocoated with an Au-Pd alloy to improve electron conduction.

Copper quantification of the released ions from the composite plates in an aqueous medium (15 mL deionized water) was determined using an atomic absorption spectrophotometer (Varian SPECTR AA-250 plus, Varian inc., Mulgrave, Victoria, Australia) at a wavelength of 324.4 nm that corresponds to copper. A calibration curve was prepared using multielemental standards (high-purity standards) at different concentrations (ppm).

Thermal stability of the iPP/CuNPs composites was determined by thermogravimetric analysis (TGA) using a TA Instruments Discovery TGA 5500 device (TA instruments Inc., New Castle, DE, USA). The analysis consisted of a temperature sweep from 25 to 600 °C under N_2_ atmosphere of a 20 mg sample with a heating rate of 10 °C/min. From 600 to 700 °C the N_2_ atmosphere was replaced by an oxygen atmosphere to assure full combustion of the residues.

Thermal transitions (melting and crystallization) of the iPP/CuNPs composites were studied by differential scanning calorimetry analysis (DSC) using a TA Instruments Discovery DSC 2500 device. Heating and cooling cycles were performed at a rate of 10 °C/min under an inert atmosphere using an N_2_ flow of 50 mL/min.

Dynamical-mechanical thermal analysis (DMTA) was used to characterize the viscoelastic behavior of the iPP/CuNPs composite as a function of temperature. A TA Instruments DMA Q800 device was employed for the analysis using a single cantilever clamp. The temperature range was from 25 to 180 °C, with a heating rate of 5 °C/min at a frequency of 1 Hz and an oscillation amplitude of 20 μm. The storage or elastic modulus (E’) was determined and reported at 30 °C.

Antibacterial activity of iPP/CuNPs composites was determined according to methods reported in ASTM E2149-01. *Pseudomonas aeruginosa* Gram (-) and *Staphylococcus aureus* Gram (+) bacteria were studied. Preparation of the bacterial suspension consisted of activation and growth of the bacterial inoculum in sterile Ringer’s solution at optimal conditions (37 °C for 16 h) until a final concentration (after dilution) of 1 × 10^5^ CFU/mL was reached.

The procedure to analyze the antibacterial activity of the composites is described as follows. First, it was conducted the addition of 25 mL of the bacterial suspension (1 × 10^5^ CFU/mL) together with the composite sample into a 250 mL flask (the composite plate was previously sterilized with UV light for 25 min); the flask was placed in an incubator at 37 °C with stirring. After the established contact time has elapsed, a sample of 0.1 mL was taken from the flask and was mixed in an Eppendorf tube with 0.9 mL of Ringer’s solution. After, a sample of 0.1 mL was taken from this mix and was placed in a polystyrene Petri dish together with sterile nutrient agar. The Petri dishes were inoculated in triplicate with the bacterial solution at different contact times (0, 2, 4, 6, and 24 h) and were kept in an incubator at 37 °C for a time interval between 18 and 24 h.

Antibacterial activity of the composites was calculated using Equation (1) [26,36]

(1) $$Antibacterial\;activity\;\left(\%\right)=\frac{Co-C}{Co}\times 100$$

where *C_o_* is the number of formed CFU (colony-forming unit) in the control dish (bacterial solution without sample) and *C* is the number of formed CFU in the dishes where the bacterial solution was in contact with the sample.

## 3. Results and Discussion

### 3.1. Wide Angle X-ray Diffraction (WAXD)

WAXD analysis was performed on the iPP/CuNPs composites and the unfilled polymer (iPP). Through this analysis, it was possible to determine the type of copper particle resulting in the processing of the composites.

**Figure 1:**
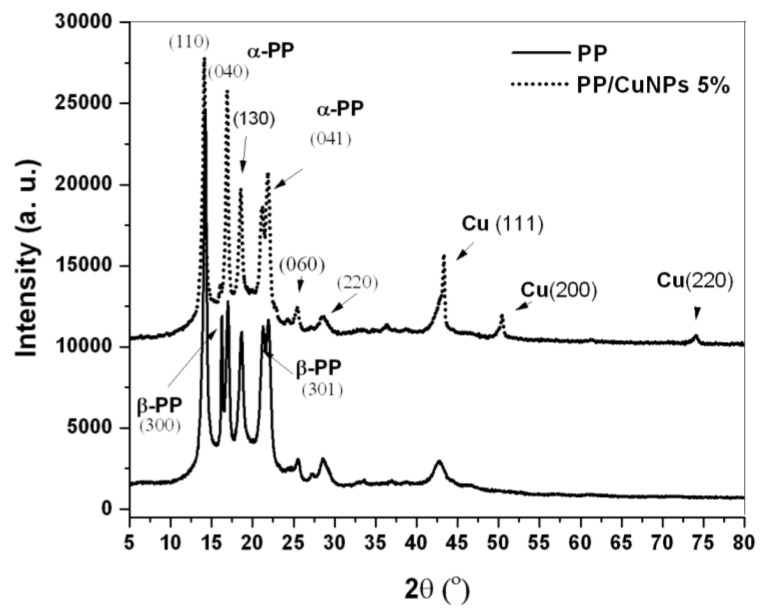
Diffractograms of iPP and PP/CuNPs 5 wt % composite.

X-ray diffractograms of iPP and a sample representative of the composites identified as iPP/CuNPs 5% (5.0 wt % of CuNPs in iPP) are shown in Figure 1. Diffractogram corresponding to iPP shows characteristic reflections of the crystals β-PP of this polymer such as 2θ = 14.1, 16.9, 18.5, 21.2, 22.1, 25.5, and 28.5° associated with the (110), (040), (130), (111), (041), (060), and (220) planes, respectively. Other reflections associated with β-PP crystals are observed at 2θ = 16.2 and 21.2° corresponding to planes (300) and (301), respectively. Additionally, a peak at 2θ = 42.8° is appreciated, suggesting the presence of nucleating agents to induce the formation of β-crystals in the commercial iPP [37,38,39].

The composite diffractogram shows some differences regarding that of the iPP; peaks intensity changes and even the peak at 2θ = 16.2° that corresponds to β-PP crystals disappears [37]. The modifications of the characteristic diffractogram of iPP can be associated with a nucleating effect of the CuNPs. Some authors have reported that the processing conditions used for preparing the CuNPs/iPP composites promoted a transformation of β-PP crystals to α-PP crystals, which is feasible too [38]. Another important change that can be observed is the presence of three new peaks associated with metallic copper (face-centered cubic structure, FCC). These peaks were found at 2θ = 43.6, 50.7, and 74.6°, corresponding to planes (111), (200), and (220), respectively [40,41,42,43].

The diffractograms of the composites did not show evidence of the formation of Cu_2_O and CuO. According to this analysis, it can be inferred that the oxidation of metallic copper during the iPP/CuNPs composite processing stage is avoided due to the coating based on nitrogenated ligands (PEI and GABA) formed over the CuNPs surface. This fact is important since in other works that report the preparation of polymeric composites based on CuNPs it is common to detect the oxidation of the CuNPs during the processing of the composite due to the strong tendency of metallic copper to form copper oxide under ambient conditions [36].

### 3.2. SEM Analysis

**Figure 2:**
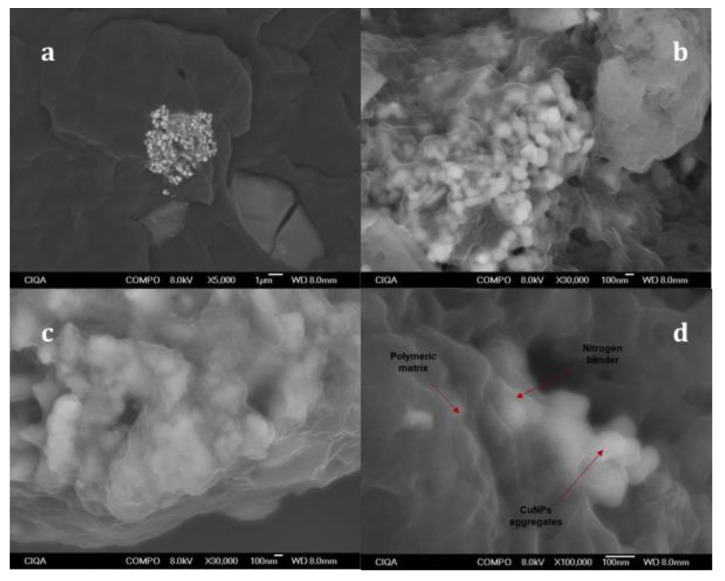
SEM images with different magnification (**a**) 5000, (**b**) 30,000, (**c**) 30,000, and (**d**) 100,000 times of sample iPP/CuNPs 0.25 wt %.

CuNPs dispersion in the polymeric matrix was analyzed by SEM micrographs. The composite samples were cryogenically fractured and coated with gold/platinum to allow electron conductivity. Figure 2 shows the micrograph of the iPP/CuNPs composite with 0.25 wt % of nanoparticles. The images were taken at different magnification levels with backscattered electron techniques or Z-contrast; this technique is able to differentiate individual heavy atoms from lighter [44].

The polymer matrix can be detected in dark gray, while the CuNPs aggregate is observed in a bright white tone with a semispherical morphology. The analysis of the high-resolution micrographs determined the presence of irregularly shaped aggregates of an approximate size of 350 nm. These aggregates are formed of subparticles with an average particle size of 36–226 nm; the addition of 0.25 wt % of CuNPs increased the particle size compared to the original (27.0 nm). This behavior is known, CuNPs increase in size when they are mixed with a polymer in the molten state and high concentrations of copper lead to larger particle sizes [29].

It is noted a different tonality in the edges of the copper nanoparticles compared to their inner part. This tonality difference can be attributed to the nitrogenated ligand, which is approximately 7 nm thick.

### 3.3. TGA Analysis

**Figure 3:**
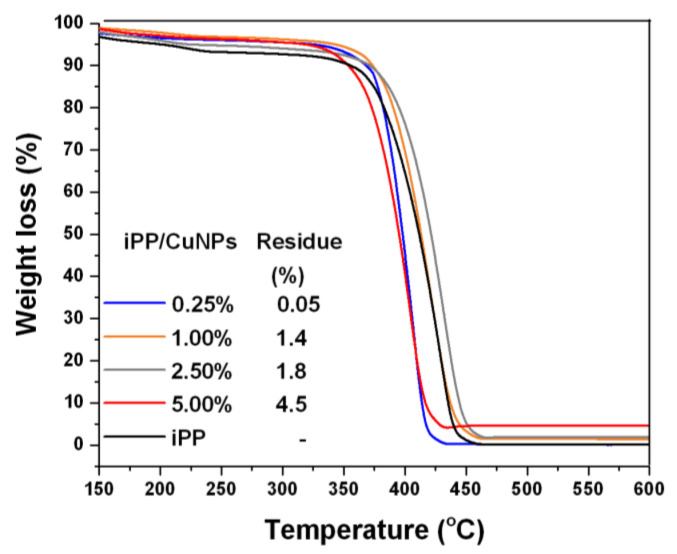
TGA thermographs of iPP and iPP/CuNPs composites.

The thermal stability of the iPP and iPP/CuNPs composites, as a measure of resistance to thermal degradation, was evaluated using TGA. Figure 3 shows TGA thermograms of weight loss as a function of the temperature of iPP and iPP/CuNPs composites.

**Figure 4:**
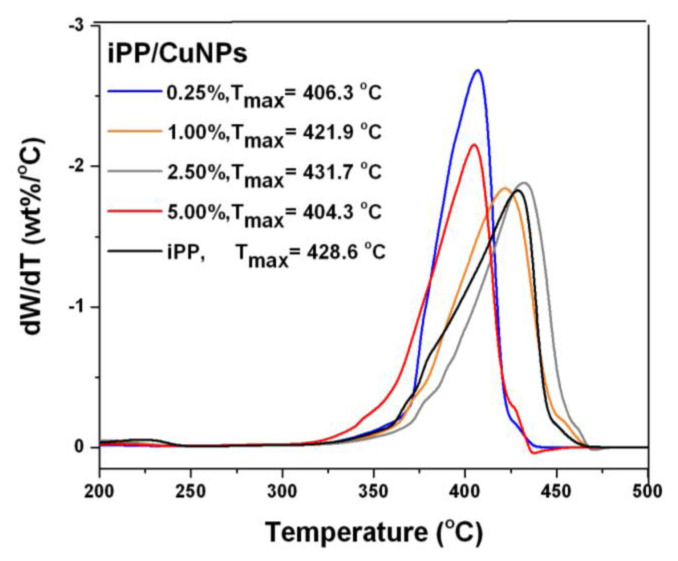
DTG curves of iPP and CuNPs/iPP composites.

All composites show greater weight loss regarding the iPP sample in the temperature range of 150–320 °C. The composites with 0.25, 1.0, and 5.0 wt % presented a lower maximum degradation temperature (T_max_) compared to the iPP that can be observed between 320 and 470 °C. The composite with 2.5 wt % shows the T_max_ at 431.7 °C, slightly higher compared to the iPP. Figure 4 illustrates the first derivative of TG curves (DTG) that confirm the stability of the samples.

The effect produced by copper on the stability of the polymeric matrix of iPP is not clear, reports indicate that the stability is negatively and positively affected, generally the positive effects are observed when using NPs of large size (850 nm) and CuO NPs [28].

The weight percentage observed at temperatures higher than 460 °C can be attributed to inorganic residues that cannot be degraded at this range of temperature. In this case, these residues can be associated with CuNPs. Residues values of the composite samples do not correspond to those used in the formulation for CuNPs. These variations can be attributed to a non-homogeneous distribution of the CuNPs in the iPP matrix.

### 3.4. DSC and DMA Analyses

The thermal transitions of the iPP/CuNPs composites at several nanoparticle concentrations were studied through DSC analysis. Besides, storage module E’ at 30 °C was obtained from the DMTA analysis for these same samples. Table 1 lists the crystallization temperature (T_c_), enthalpy of crystallization (ΔH_c_), melting temperature (T_m_), enthalpy of melting (ΔH_m_), the crystalline fraction (X_c_), and the storage modulus (E’) of the iPP/CuNPs composites.

Thermal transition data show growth with the increase of the filler in the iPP matrix, except for the composite with 1.0 wt % copper. This can be attributed to a non-homogeneous distribution of the NPs. The T_m_ was slightly lower compared to the unfilled iPP, while the T_c_ increased with the addition of CuNPs to the iPP. An increase of 5 °C was observed in the composite with 5.0 wt %. Yanying Jiang et al. observed a rise of 3 °C in the T_c_ of iPP/CuO composites. They attributed this increase to the low efficiency of CuONPs as nucleating agents [28]. The greater increase in T_c_ observed in the iPP/CuNPs composites suggests that the CuNPs have a greater nucleating effect on the crystallization rate of iPP compared to CuONPs.

The crystalline fraction (X_c_) was calculated using the formula X_c_ = ΔH_m_/ΔH_f_ (100%). The enthalpy of melting for a 100% crystalline iPP was taken from the literature as ΔH_f_ 100% = 209 J/g [45]. In most of the analyzed samples, the CuNPs in the polymeric matrix caused an increment in the crystalline fraction (X_c_). The increment of the crystalline fraction can be associated with a nucleating effect of the CuNPs on the iPP matrix. As crystallization is an exothermic process, then the copper particles can absorb and dissipate fast the heat generated by nearby polymer chains. This effect can cause the iPP chains closest to the copper particle’s surface to show a faster crystallization rate. Other authors who consider that metallic nanoparticles can induce a nucleating process during the crystallization of the polymeric matrix have reported a similar explanation [46,47]. They point out that the high thermal conductivity of polymers that contains metal nanoparticles is evidence supporting this idea. However, it must be considered that copper nanoparticles do not always show the same behavior since it depends on several factors, such as the oxidation state, size, and functionalization, among others, so these reports should be taken with caution.

A comparison of the results of X_c_ and storage module E’ indicates a good correlation. The iPP/CuNPs composites that showed higher X_c_ values compared to those obtained for iPP had better E’ modulus values. These CuNPs composites samples showed E’ values between 2700 to 4000 MPa that were superior compared to unfilled iPP that had an E’ value of 1764 MPa. An increase in E´ values shows a growth in the rigidity of the composite and reinforcement of the polymeric matrix, which was less significant at high concentrations of copper. By increasing the number of CuNPs in the polymer matrix, the formation of aggregates is favored, therefore, more defective points will also be produced in the matrix [48]. Aggregate formation in CuNPs composites seems inevitable even at concentrations of 0.25 wt % (Section 3.2). The functionalization of CuNPs is not enough to avoid the aggregation of particles. New research involving the use of compatibility agents for iPP and functionalized CuNPs should be carried out to contribute to the synthesis of iPP composites with high copper concentration.

In general, the data of X_c_ and storage module E´ agree to expected values. J. A. Molefi et al. reported a similar behavior when studying polyethylene composites with CuNPs [48]. In this work, the E´ values increase with rising copper concentration. The highest E´ values are achieved at copper concentrations of 1 vol.%, the E´ values were very similar to concentrations of 3 and 5 vol.%. This increase is attributed to several reasons, such as weak interfacial bonding between the CuNP and matrix interfaces, aggregates of Cu nanoparticles, and nanoparticle processing-related defects [48].

### 3.5. Cupric Ion Release from PP/CuNPs Composites

**Figure 5:**
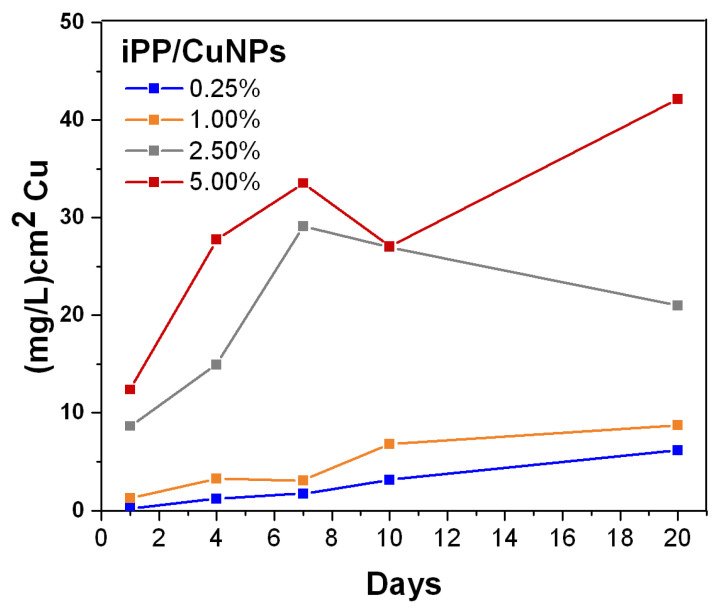
Concentration of released copper from iPP/CuNPs composites.

It is known that the antibacterial mechanism of these sorts of composites is based on the release of metal ions in the presence of moisture and oxygen in the air [49]. In this work, the release of copper ions from the iPP/CuNPs composites was determined by atomic absorption analysis. Figure 5 contains the plots of copper concentration in mg/L*cm^2^ released in deionized water as a function of exposition time (number of days) for the different iPP/CuNPs composites (0.25, 1.0, 2.5, and 5.0 wt %).

In general, it can be appreciated a raise in the copper ions release when CuNPs concentration increases. This result suggests that an increment in the crystalline fraction of the composite (see Table 1) did not affect the copper ion release capability. There was also an increase in copper concentration as the exposure time of the sample in water increased from 1 to 10 days. After the tenth day, it is not observed a significant increment in the copper concentration. The highest amount of released copper was obtained with the iPP/CuNPs composite with 5 wt % of nanoparticles, which was 12.5 and 27 mg/L*cm^2^ at 1 and 10 days, respectively. These results are in agreement with those reported by other authors, as described below.

Tamayo et al. reported a copper ions concentration of 18 and 26 mg/L*cm^2^ at 1 and 10 days, respectively, for a composite with 5.0 wt % of CuNPs in polyethylene produced by in situ polymerization [50]. Palza et al. reported copper concentrations of 22 and 80 mg/L*cm^2^ at 1 and 10 exposition days corresponding to an iPP/CuNPs composite at 5.0 wt % [30]. These composites were obtained by a melt mixing method applying an additional treatment, which could be: (a) a predispersion step of the CuNPs in ethanol, (b) preparation of a nanoparticle masterbatch with a compatibilizer, or (c) CuNPs functionalization.

It should be noted that the first hours of contact of the sample with the microorganism are relevant for the efficacy of the antibacterial activity. In the case of the composite iPP/CuNPs at 5.0 wt % that showed a copper concentration of 12.5 mg/L*cm^2^ at the first contact day, it would suggest that this composite should show a good antibacterial efficiency.

### 3.6. Antibacterial Activity

Table 2 shows the required contact time of the sample with the bacterial suspension to reach different antibacterial activity percentages by the iPP/CuNPs composites toward *P. aeruginosa* and *S. aureus* bacteria.

For all composites, it was observed a higher antibacterial activity toward *P. aeruginosa* with respect to *S. aureus*. This behavior could be associated with differences in the cell wall of the bacteria. As has been reported by several studies, Gram (-) bacteria are more susceptible to metal ions compared to Gram (+) bacteria [19,50,51,52].

The composite iPP/CuNPs at 5.0 wt % of nanoparticles in iPP showed the highest antibacterial activity. It required only 2 h to achieve total antibacterial activity (100%) toward *P. aeruginosa* and 4 h toward *S. aureus.* The composite with 2.5 wt % of CuNPs in iPP also achieved total antibacterial activity toward *P. aeruginosa* at 2 h of contact and toward *S. aureus* at 6 h of contact.

It is important to mention that the iPP/CuNPs composite at 5.0 wt % showed higher antibacterial activity compared to other similar works reported in the literature. According to España et al., composites based on iPP charged with 5.0 wt % of commercial CuNPs, which were treated with plasma to increase its hydrophilicity, achieve 100% antibacterial activity at 3 h of contact toward *P. aeruginosa* and 98% at 6 h toward *S. aureus* [36]. The treatment with plasma improves the antimicrobial activity but it can reduce the mechanical properties of the composite. In another report, Palza et al. evaluated PP/CuNPs composite with 5.0 vol.% of nanoparticles, achieving antibacterial activity of 99.8% toward *P. aeruginosa* and *S. aureus* after 60 min of contact [25]. However, the quantity of CuNPs is too high, since 5.0 vol.% equals 36 wt %. This conversion agreed with the calculations used by other reports to show the equivalence between wt % with vol.% in their copper/iPP composite formulations [47,53,54,55].

Another important aspect that must be considered in antimicrobial activity studies is the chemical nature of the copper nanoparticles. It is known that CuNPs chemically stable to oxidation have higher antimicrobial activity and less cytotoxicity compared to CuO and Cu_2_O nanoparticles. This suggests that CuNPs/iPP composites obtained in the present research work could be materials with low toxicity maintaining a high antimicrobial activity for a longer lifetime [56].

## 4. Conclusions

The CuNPs preserved its metallic state (no oxidation) during the processing of the composites because of the functionalization of the nanoparticles with the nitrogenated ligands. Additionally, the hydrophilic character of these functionalized nanoparticles improved the copper ions release from the composite in an aqueous medium.

The highest amount of released copper was obtained with the composites that contained 2.5 and 5 wt % of the CuNPs in the iPP matrix. Composites’ capability to release copper ions agreed with the antibacterial properties; composites with 2.5 and 5.0 wt % of CuNPs show full antibacterial activity (100%) toward *P. aeruginosa* after 2 h of contact and toward *S. aureus* after 4 h of contact.

The presence of CuNPs in the polymer matrix slightly decreased the thermal stability but improved the crystallinity and storage modulus. The copper/polymer composites could be an excellent alternative to be employed as antimicrobial materials, but it is necessary to do more detailed studies about its toxicity. The composites with the highest antibacterial activity are potential materials for wastewater disinfection, packaging, and biomedical products.

## Figures and Tables

**Table 1:** DSC and DMA data corresponding to iPP/CuNPs composites.

Samples	T_c_ ^a^ (°C)	ΔH_c_ ^a^ (J/g)	T_m_ ^a^ (°C)	ΔH_m_ ^a^ (J/g)	X_c_ ^b^	E’ ^c^ (MPa)
PP	117.1	93.7	164.2	92.4	0.44	1764
PP/CuNPs 0.25%	118.9	99.3	163.7	97.8	0.47	3983
PP/CuNPs 1.00%	118.6	90.4	162.5	87.9	0.42	1695
PP/CuNPs 2.50%	121.1	93.4	163.6	93.5	0.45	2839
PP/CuNPs 5.00%	122.1	93.9	163.5	96.7	0.46	2699

a—Obtained by DSC, b—determined as X_c_ = ΔH_m_/209 J/g, and c—obtained by DMA at 30 °C.

**Table 2:** Antibacterial activity (AA) of iPP/CuNPs composites toward.

Composite PP/CuNPs	*P. aeruginosa* Gram (-)	*S. aureus* Gram (+)
	AA(%)/h	AA(%)/h
0.25%	53/24	76/24
1.00%	100/24	98/24
2.50%	100/2	100/6
5.00%	100/2	100/4

*P. aeruginosa* and *S. aureus* bacteria.

## Data Availability

The data presented in this study are available on request from the corresponding author.
